# Racial effects on masimo pulse oximetry: impact of low perfusion index

**DOI:** 10.1007/s10877-023-01113-2

**Published:** 2024-01-19

**Authors:** Vikrant Sharma, Steven J. Barker, Rebecca Sorci, Linus Park, William C. Wilson

**Affiliations:** 1 Vice President, Optical Sciences, Masimo Corp, Irvine, CA USA; 2 Chief Science Officer, Masimo Corp, Irvine, CA USA; 3 Director, Clinical Publication Review and Communications, Masimo Corp, Irvine, CA USA; 4 Vice President Regulatory Affairs, Masimo Corp, Irvine, CA USA; 5 Executive Vice President, Clinical Research Operations and Medical Affairs, Masimo Corp, Irvine, CA USA

**Keywords:** Pulse oximetry, Oxygen saturation, Race, Ethnicity, Skin pigmentation, Occult hypoxemia, Peripheral perfusion

## Abstract

**Purpose:** Evaluate the SpO_2_-SaO_2_ difference between Black and White volunteer subjects having a low perfusion index (Pi) compared to those having a normal Pi. **Methods:** The Pi data were abstracted from electronic files collected on 7183 paired SpO_2_-SaO_2_ samples (3201 Black and 3982 White) from a recently reported desaturation study of 75 subjects (39 Black and 36 White) where SaO_2_ values were sequentially decreased from 100 to 70%. The Pi values from that dataset were divided into two groups (Pi ≤ 1 or Pi > 1) for analysis. A Pi value ≤ 1 was considered “low perfusion” and a Pi value > 1 was considered “normal perfusion”. Statistical calculations included values of bias (mean difference of SpO_2_-SaO_2_), precision (standard deviation of the difference), and accuracy (root-mean-square error [A_RMS_]). During conditions of low perfusion (Pi ≤ 1, range [0.1 to 1]), overall bias and precision were + 0.48% ± 1.59%, while bias and precision were + 0.19 ± 1.53%, and + 0.91 ± 1.57%, for Black and White subjects, respectively. **Results:** During normal perfusion (Pi > 1, range [1 to 12]), overall bias and precision were + 0.18% ± 1.34%, while bias and precision were -0.26 ± 1.37%, and − 0.12 ± 1.31%, for Black and White subjects, respectively. A_RMS_ was 1.37% in all subjects with normal perfusion and 1.64% in all subjects with low perfusion. **Conclusion:** Masimo SET® pulse oximeters with RD SET® sensors are accurate for individuals of both Black and White races when Pi is normal, as well as during conditions when Pi is low. The A_RMS_ for all conditions studied is well within FDA standards. This study was conducted in healthy volunteers during well-controlled laboratory desaturations, and results could vary under certain challenging clinical conditions.

## Introduction

Clinical conditions resulting in low peripheral perfusion are a recognized confounder of conventional pulse oximetry [1–3]. Early reports comparing the performance of different pulse oximeters during low perfusion demonstrated a variability in oxygen saturation (SpO_2_) accuracy between device brands [1, 2]. Some manufacturers have features to address low perfusion, such as indicators when pulse oximetry signals are not adequate. Masimo accounts for the potential errors resulting from low perfusion and other common confounders (e.g., motion) by developing advanced engineering design solutions and signal-processing techniques known as Masimo Signal Extraction Technology® (SET®). Recent reports suggested that pulse oximeter performance during poor perfusion can still vary amongst present-day device brands [4].

The effect of skin tone, and therefore race, on SpO_2_ accuracy is another topic of longstanding interest [5, 6]. Increased emphasis has been focused on this important subject since the 2020 publication by Sjoding, et al. [7]. In this report, investigators combined data from all pulse oximeter manufacturers together and retrospectively surveyed results from ICU patients across 178 hospitals, allowing as much as 10 min between the time stamps for SpO_2_ values measured by pulse oximetry and arterial blood gas saturation (SaO_2_) measurements made by CO-oximetry. The results showed a tendency for the SpO_2_ to read higher than SaO_2_ to a greater extent in Black patients than in White patients [7]. Furthermore, “occult hypoxemia”, defined by the investigators as a SpO_2_ reading 92–96% when the SaO_2_ was < 88%, was three times more common in Blacks than in Whites in this study [7]. Others found similar tendencies [8–19], but to less of an extent than that found by Sjoding, et al.

In response to these reports, we conducted a focused evaluation of the accuracy of Masimo SET® pulse oximeters with RD SET® sensors on healthy Black and White volunteers undergoing controlled desaturation studies in the laboratory [20]. The results revealed that Masimo SET® pulse oximeters deliver accurate values across the skin tone range, as the statistical bias (mean difference of SpO_2_-SaO_2_) and precision (standard deviation of difference) were − 0.20 + 1.40% for Black and − 0.05 + 1.35% for White subjects, and occult hypoxemia was rare and did not occur in Black subjects.

Recently, data from a well-known and longstanding pulse oximeter testing laboratory were reported in a non-peer reviewed preprint paper, which raised new concerns regarding the potential combined confounders of low perfusion coupled with dark skin pigmentation resulting in greater differences between SpO_2_ and SaO_2_ [21].

In light of these concerns, we performed a secondary analysis of the dataset presented in our recently published study on Black and White subjects undergoing controlled desaturations. The goal of this subgroup analysis is to assess the accuracy of Masimo SET® pulse oximetry with RD SET® sensors during conditions of normal and low perfusion for both Black and White subjects.

## Methods

The Perfusion Index (Pi) data were retrospectively abstracted from electronic files collected during a laboratory desaturation study conducted between September 2015 and July 2021 and recently published in this journal [20].

The Pi is the measured ratio of pulsatile to the non-pulsatile signal in the plethysmography waveform of the infrared light emitting diode (LED). Masimo devices support a Pi ranging from 0.02 to 20%, as a non-invasive measure of the pulse strength of arteriolar blood volume interrogated by the pulse oximeter sensor. Pi is clearly related to peripheral tissue perfusion [22], but it also depends on other variables that affect local arteriolar volume, including vessel compliance [23].

This study analyzed 7183 paired SpO_2_-SaO_2_ samples (3201 Black and 3982 White) from 75 subjects (39 Black and 36 White) collected during a desaturation protocol wherein SaO_2_ values were sequentially decreased to obtain six stable plateau values between 100 and 70%. During each stable plateau, arterial blood gas (ABG) samples were obtained from a radial arterial cannula, and simultaneous SpO_2_ and Pi readings were recorded using Masimo RD SET® sensors (Masimo Corporation, Irvine, California). The ABG samples were analyzed on a Radiometer ABL-835 Flex CO-Oximeter (Radiometer Inc., Brea, California). The protocol included subject warming of the upper extremities and/or torso away from the pulse oximeter site. In addition, no subjects were actively cooled to decrease peripheral perfusion or Pi. The protocol was consistent with the ISO 80601-2-61 pulse oximetry standard and underwent review and approval by the Institutional Review Board of Ethical & Independent (E&I) Review Services (Lee’s Summit, MO).

**Fig. 1:**
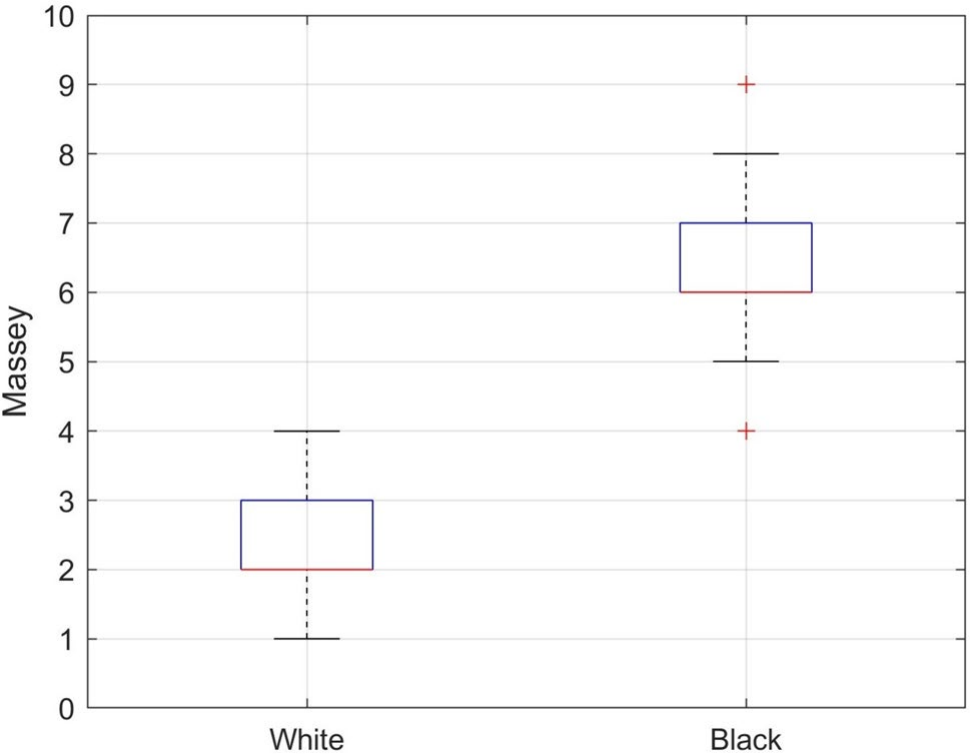
Box plots showing the interquartile range (IQR) of Massey Scale values for Black and White subjects. The box plot includes a horizontal red line within each box representing the median; the top and bottom of each box represent the upper and lower limits of the IQR, and the whiskers represent the minimum and maximum values (excluding outliers shown with red + symbol). (Color figure online)

Subjects who self-identified as Black had Massey Scale values ranging from 4 to 9 (median 6, interquartile range [IQR] 6–7), while those who self-identified as White ranged in Massey Scale values from 1 to 4 (median 2, IQR 2–3). Box plots representing the IQR of Massey Scale values for Black and White subjects are shown in Fig. 1, which show that the analysis was conducted on two distinct pigment groups. There was a median of 72 paired samples per subject for the Black population and 96 per subject for the White population. Additional details about the demographics, methodology, and results of the initial investigation can be found in the 2023 published study [20].

**Fig. 2:**
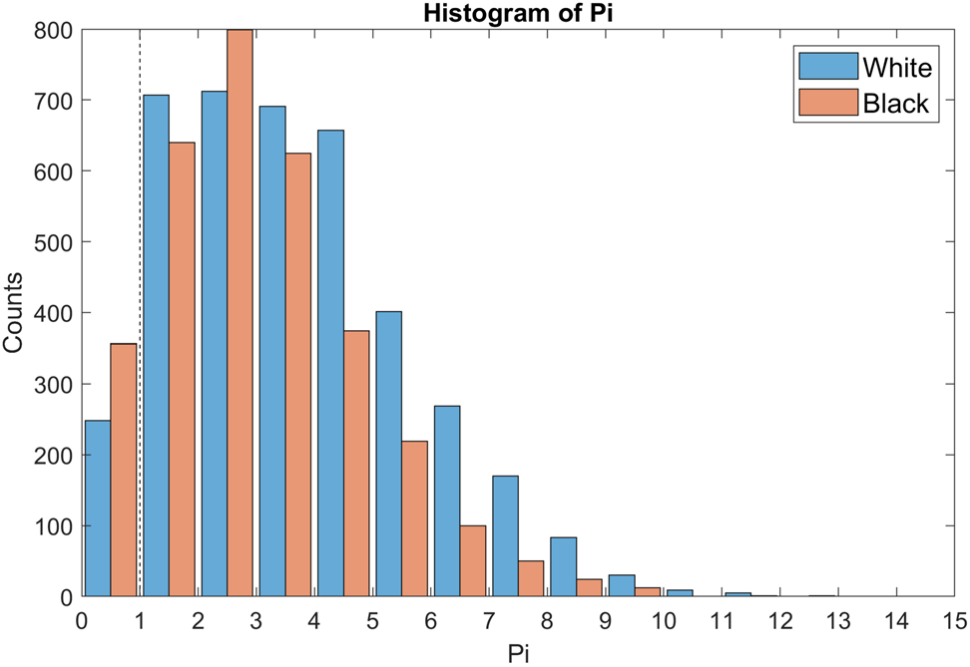
Pi distribution histogram (sample count on y-axis, Pi on x-axis) from self-identified Black (salmon tint bars) and White (blue tint bars) subjects. (Color figure online)

**Fig. 3:**
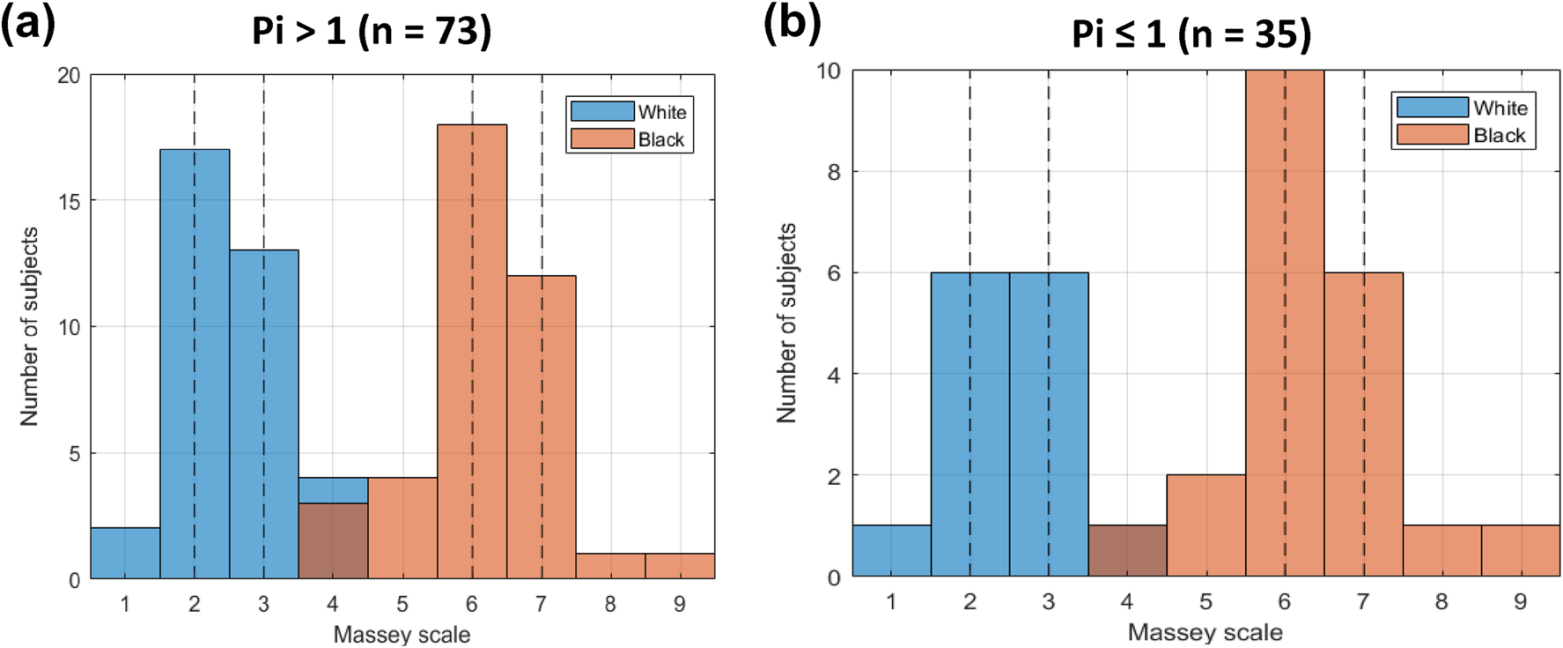
Massey Scale distribution histogram from self-identified Black (salmon tint bars) and self-identified White (blue tint bars) subjects contributing data to the Pi > 1 (Fig. 3a) and Pi ≤ 1 (Fig. 3b) groups. The vertical dashed lines indicate the IQR for each group. (Color figure online)

For this secondary evaluation, data are grouped by self-declared race (Black vs White) and perfusion status (Pi ≤ 1 or Pi > 1) for analysis. A Pi value ≤ 1 is considered “low perfusion” and a Pi value > 1 is considered “normal perfusion”. These values are comparable to the Pi thresholds for normal and low perfusion determined in previous studies [21, 22, 24]. The distribution of Pi values in this study is shown in Fig. 2. The Massey Scale distribution is further characterized by displaying the histogram of subjects contributing to the normal and low perfusion groups in Fig. 3a and b, respectively. Of the 75 subjects in the study, 73 subjects (37 Black and 36 White) contributed data to the normal perfusion group, while 35 subjects (21 Black and 14 White) contributed data to the low perfusion group. Note that 33 subjects had both normal and low perfusion data pairs obtained during different sample collections.

Statistical calculations include values of bias (mean difference of SpO_2_-SaO_2_), precision (standard deviation of the difference), and accuracy (root-mean-square error [A_RMS_]). Since the blood gas sampling procedure used replicates nested within each subject, the precision and A_RMS_ are adjusted for repeated measures using a random effects model (MATLAB, fitlme function). This model provides equivalent results to those described in Bland and Altman [25], but it accounts for multiple sources of variation. The incidence of occult hypoxemia, defined by previous investigators as a SpO_2_ reading 92–96% when the SaO_2_ is < 88% [7], is also assessed during low perfusion conditions.

## Results

**Fig. 4:**
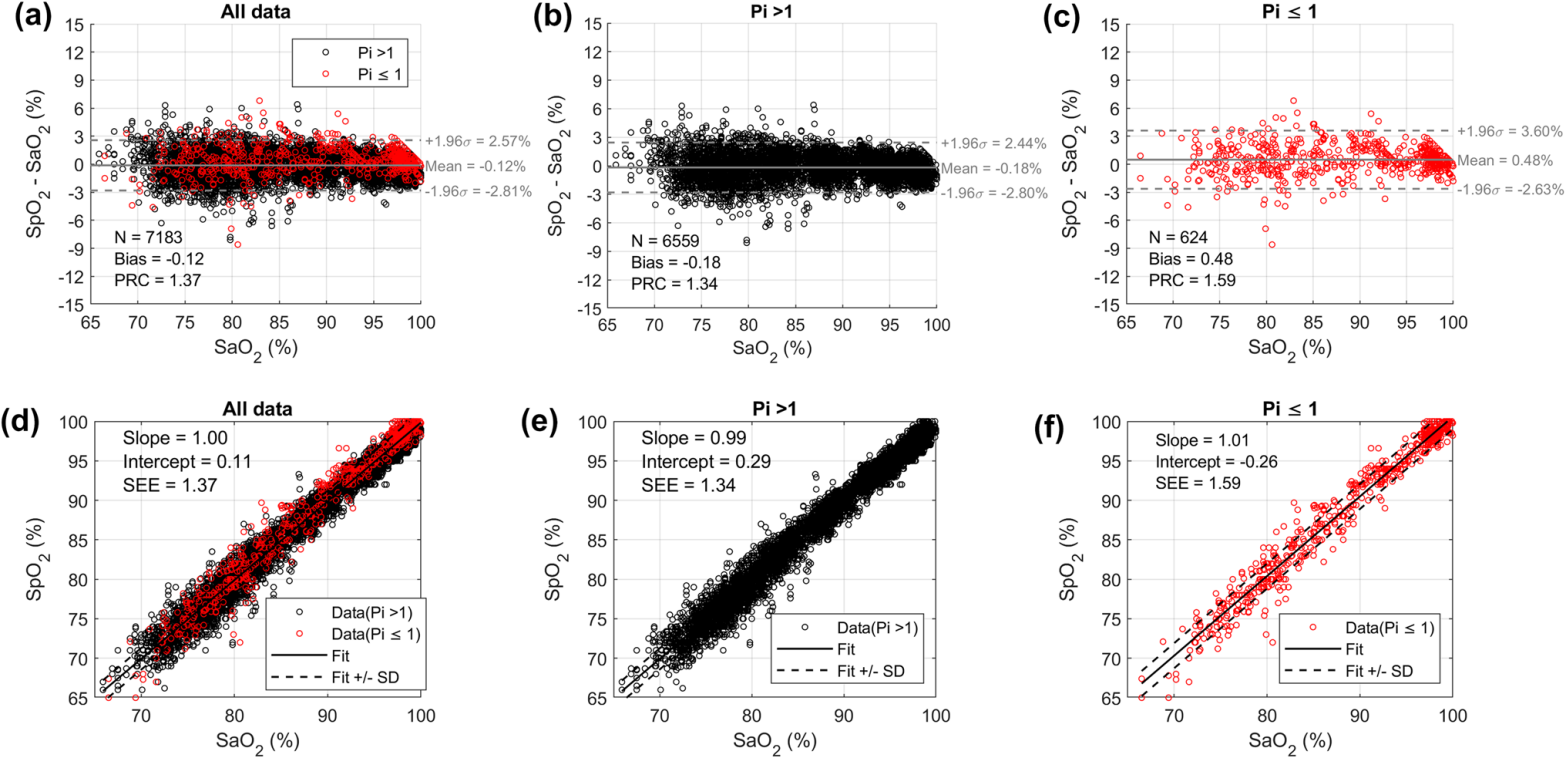
Scatter plot (SpO_2_ versus SaO_2_) along with performance metrics for all Pi data combined (a), Pi > 1 (b), and Pi ≤ 1 (c), as well as residual plot (SpO_2_-SaO_2_ versus SaO_2_) along with performance metrics for all Pi data combined (d), Pi > 1 (e), and Pi ≤ 1 (f). Red dots indicate Pi ≤ 1, black dots indicate Pi > 1. SEE = standard error estimate, Bias = mean SpO_2_-SaO_2_ difference, PRC = Precision (standard deviation of the bias). (Color figure online)

Low perfusion (Pi ≤ 1) was registered in 624 of the 7183 SpO_2_-SaO_2_ data pairs during the desaturation study, while the remaining 6,559 data pairs had normal perfusion (Pi > 1). Figure 4 shows scatter plots of SpO_2_ versus SaO_2_ measured by CO-oximeter, along with the residual plots of the SpO_2_-SaO_2_ difference versus SaO_2_, for subjects with low perfusion (red dots, Pi ≤ 1), normal perfusion (black dots, Pi > 1), as well as the combined spectrum of Pi values (all data). In the scatter plots (Fig. 4a, b and c), the solid lines show linear regression best-fits, and the dotted lines indicate the ± standard deviation of the fit. The scatter plots for subjects with Pi ≤ 1 and for subjects with Pi > 1 are visually similar. In the residual plots (Fig. 4d, e and f), the solid lines show the mean difference of SpO_2_-SaO_2_ (bias), and the dotted lines indicate the limits of agreement. Bias and precision of subjects with low and normal perfusion are comparable at + 0.48 ± 1.59% and + 0.18 ± 1.34%, respectively, with low perfusion results slightly higher. Occult hypoxemia did not occur in any subjects during low perfusion (Fig. 4f). The mean SpO_2_-SaO_2_ difference between low and normal perfusion groups is + 0.3%, and the scatter distribution showed highly overlapping error distributions for both subjects with low and normal perfusion (Fig. 4a).

The mean SpO_2_-SaO_2_ difference (bias) and precision (standard deviation of the difference) obtained from Black, White, and all (combined) subjects with both low and normal perfusion are shown in Table 1, along with the number of data pairs and subjects in each group.

**Table 1:** Tabulated summary of performance statistics for Black, White, and Overall

Masimo SET®	Black		White		All	
	Pi ≤ 1	Pi > 1	Pi ≤ 1	Pi > 1	Pi ≤ 1	Pi > 1
Bias (%)	+ 0.19	− 0.26	+ 0.91	− 0.12	+ 0.48	− 0.18
Precision (%)	1.53	1.38	1.57	1.32	1.57	1.35
A_RMS_ (%)	1.54	1.41	1.82	1.32	1.64	1.37
N_pairs_	372	2829	252	3730	624	6559
N_subjects_	21	37	14	36	35	73

A_RMS_ and precision were adjusted for repeated measures. Bias = Mean SpO_2_-SaO_2_ difference, Precision = Standard deviation of the difference, A_RMS_ = Root-mean-square error

Table 2 shows a summary of the bias, precision, and accuracy (A_RMS_) obtained in Black and White subjects with Pi ≤ 1, Pi > 1, and all (combined) data during low oxygen saturation (SaO_2_ < 90%).

**Table 2:** Tabulated summary of performance statistics for Black, White, and Overall during low arterial oxygen saturation (SaO_2_ < 90%)

	Black		White		All	
	Pi ≤ 1	Pi > 1	Pi ≤ 1	Pi > 1	Pi ≤ 1	Pi > 1
Bias (%)	+ 0.17	− 0.19	+ 0.76	+ 0.09	+ 0.41	− 0.13
Precision (%)	2.01	1.61	2.06	1.52	2.01	1.56
A_RMS_ (%)	2.01	1.62	2.20	1.52	2.05	1.57
N_Pairs_	178	1586	122	2222	300	3808
N_Subjects_	12	33	9	36	21	69

A_RMS_ and precision were adjusted for repeated measures. Bias = Mean SpO_2_-SaO_2_ difference,

Precision = Standard deviation of the difference, A_RMS_ = Root-mean-square error

**Fig. 5:**
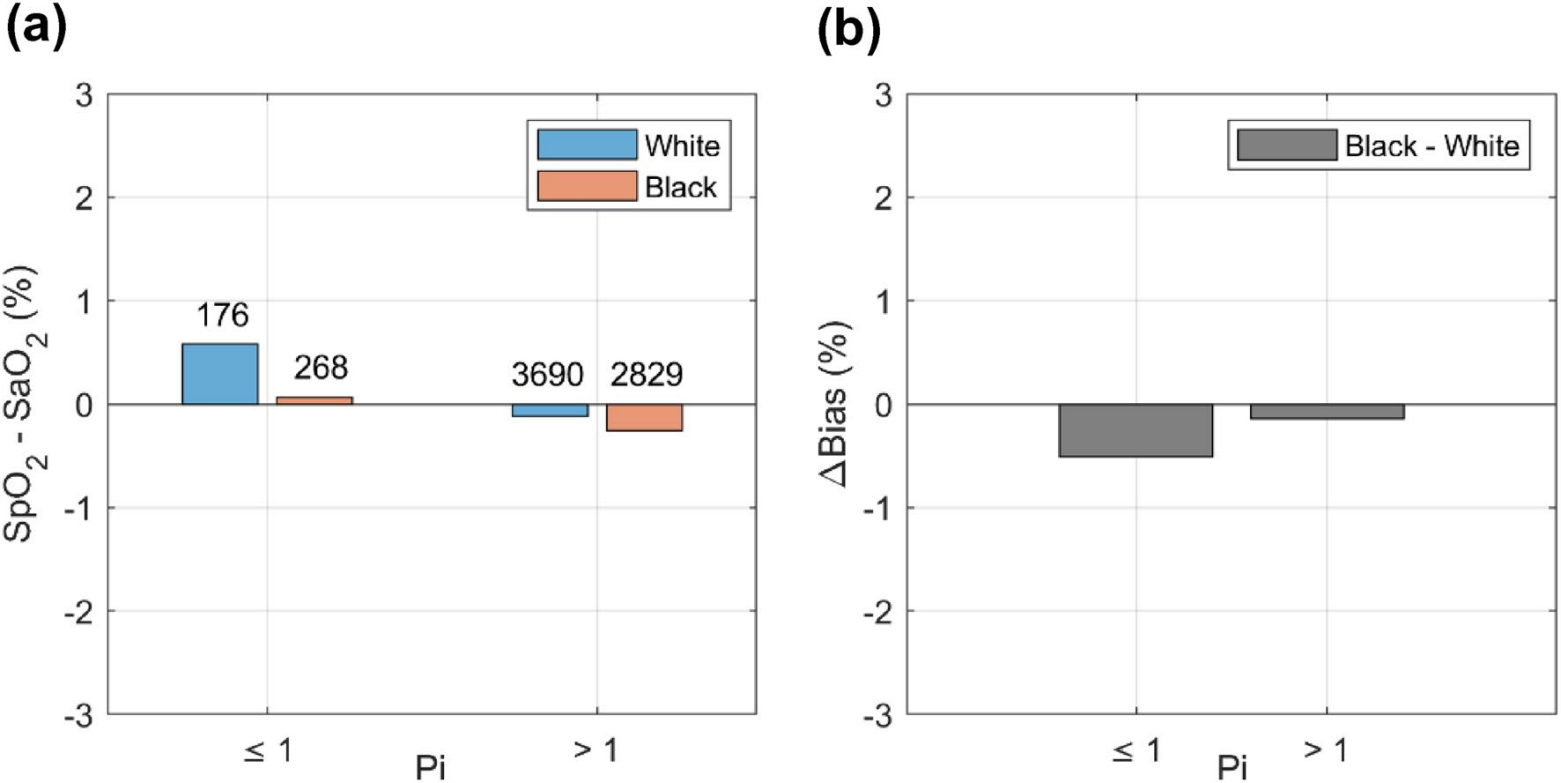
Bias (SpO_2_-SaO_2_) in Black (salmon tint bars) and White (blue tint bars) subjects with Pi ≤ 1 and Pi > 1 (Fig. 5a), and Fig. 5b similar plot showing the difference between the biases (Δ bias) of Black and White subjects. (Color figure online)

Subjects in both racial groups demonstrated a small positive bias during low perfusion (Pi ≤ 1) conditions, with White subjects experiencing greater bias (+ 0.91) than Black subjects (+ 0.19) during this condition. During normal perfusion (Pi > 1), both groups exhibited a small negative bias, with Black subjects experiencing a slightly greater bias (− 0.26) than White subjects (− 0.12). These results are graphically displayed in Fig. 5a. A plot of the difference between the biases (Δ bias) obtained for Black and White subjects at low (− 0.72) and normal (− 0.14) perfusion conditions is provided in Fig. 5b.

**Fig. 6:**
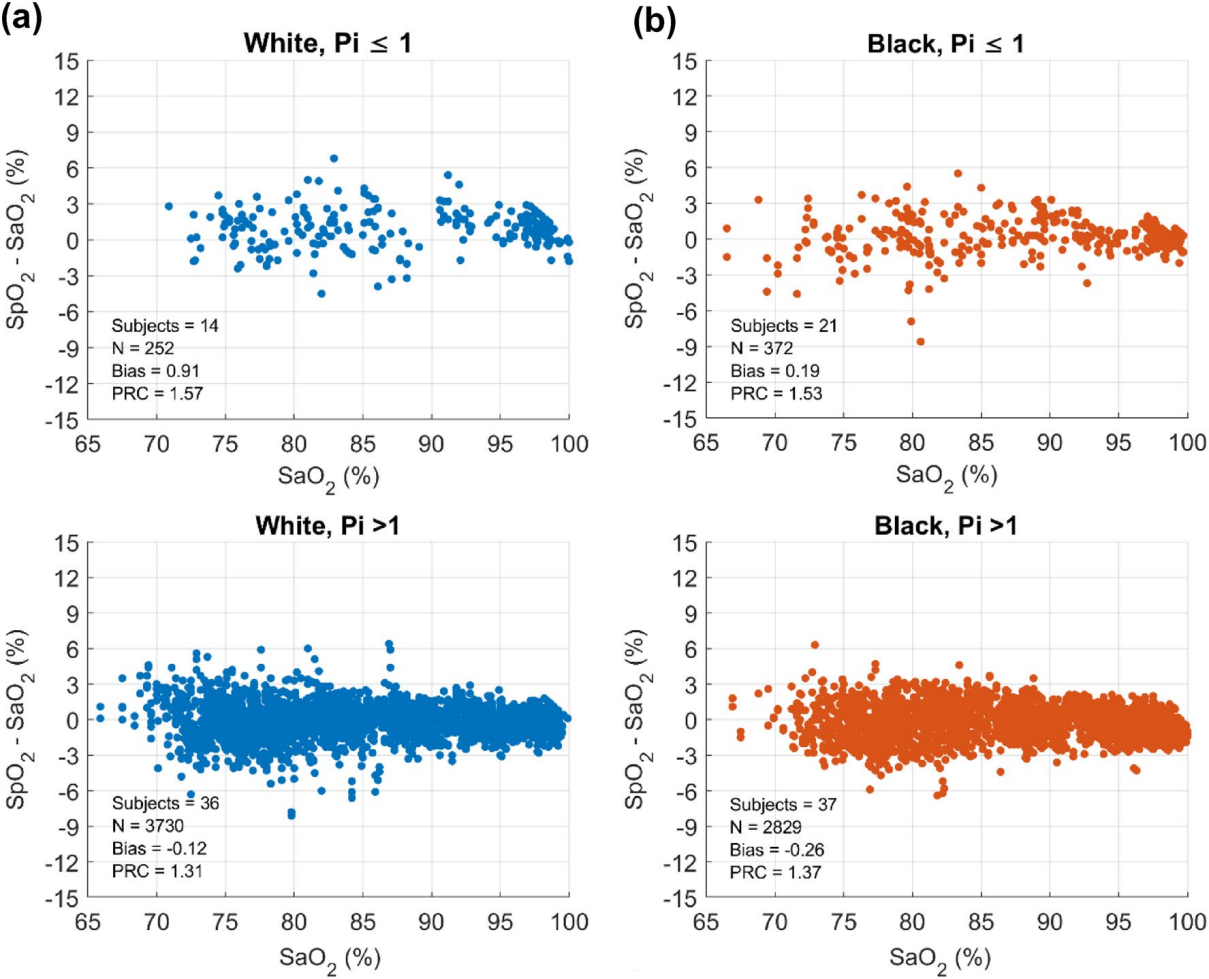
Scatter plot of SpO_2_-SaO_2_ versus SaO_2_, along with performance metrics for individual subjects with Pi ≤ 1 in White subjects (blue dots, Fig. 6a) and Black subjects (salmon dots, Fig. 6b). (Color figure online)

Scatter plots of SpO_2_-SaO_2_ (y-axis) versus SaO_2_ (x-axis) along with performance metrics for White subjects (blue dots, Fig. 6a) and Black subjects (salmon dots, Fig. 6b), are graphically displayed for both low (Pi ≤ 1) and normal (Pi > 1) peripheral perfusion groups. During low perfusion conditions, bias and precision are + 0.91 ± 1.57% for 14 White subjects (Fig. 6a) and + 0.19 ± 1.53% for 21 Black subjects (Fig. 6b), while under normal perfusion conditions, bias and precision are -0.12 ± 1.31% for 36 White subjects (Fig. 6a) and − 0.26 ± 1.37% for 37 Black subjects (Fig. 6b).

**Fig. 7:**
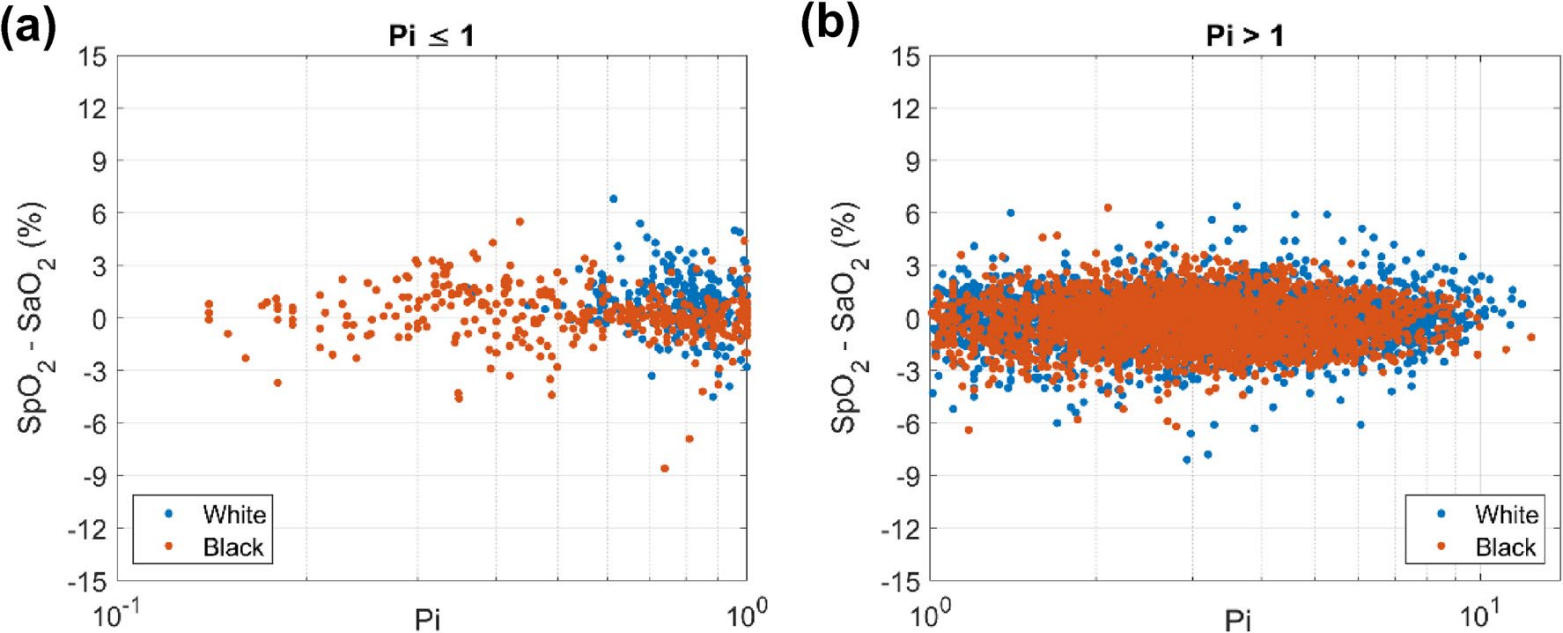
Scatter plot (SpO_2_-SaO_2_ versus Pi) for individual Black (salmon dots) and White (blue dots) subjects with Pi ≤ 1 (Fig. 7a) and Pi > 1 (Fig. 7b). (Color figure online)

Each population included some data outliers, which were attributed to instability during the desaturation procedure. However, none of the outlier data points were excluded from this analysis. Figure 7 shows scatter plots of mean SpO_2_-SaO_2_ difference versus Pi plotted on a logarithmic scale for ease of comparison in the low range. The scatter did not exhibit any systematic bias trending with lower Pi in any of the population groups. Notably, Black individuals had a lower range of Pi values, with the lowest value = 0.14%, while the lowest recorded Pi value for White individuals was 0.36%.

## Discussion

The data demonstrate that Masimo SET® pulse oximeters with RD SET® sensors are equally accurate when tested on healthy Black and White volunteers during conditions of normal as well as low peripheral perfusion. Under conditions of low perfusion (Pi ≤ 1), bias and precision were + 0.19 ± 1.53% for Black subjects and + 0.91 ± 1.57% for White subjects. In addition, the difference between the biases (mean difference of SpO_2_-SaO_2_) obtained from Black and White subjects during low perfusion was -0.71%. This difference is not likely to be relevant, because most commercially-available pulse oximeter devices display SpO_2_ only to the nearest 1%. In addition, occult hypoxemia did not occur in any subjects during low perfusion and occurred in only one data pair from a White subject during normal perfusion. Moreover, these results indicate an absence of racial differences in Masimo SET® pulse oximetry performance during conditions of poor perfusion in healthy volunteers. The overall accuracy (A_RMS_) based on entire dataset was 1.38%, 1.42% for Black, and 1.35% for White population, as reported previously [20]. During low perfusion conditions, the overall A_RMS_ in all subjects was 1.64%. Notably, there were distribution outliers in both populations that are attributed to natural instability in the desaturation plateaus. However, we did not omit any of the outliers from the dataset.

The absence of relevant differences in SpO_2_-SaO_2_ between Black and White subjects throughout the Pi spectrum can be attributed to the engineering advancements of Masimo SET® pulse oximetry, which were designed to address the confounders of motion, low perfusion and skin pigment, as well as the calibration and validation paradigm utilized by Masimo. The U.S. FDA Guidance for medical-grade pulse oximeters requires only a minimum of two subjects, or 15% of the study pool, to have dark skin pigmentation during validation studies for 510(k) clearance. However, for over two decades, Masimo has adhered to a more stringent standard for evaluating the impact of skin pigment by calibrating and validating the performance of its devices using nearly equal numbers of dark skin and light skin individuals. The results of this study demonstrate that Masimo SET® technology is effective in minimizing the impact of low perfusion and skin pigment, enabling Masimo pulse oximeter devices to measure SpO_2_ accurately and reliably across the spectrum of perfusion conditions and skin tones.

The recent preprint manuscript from Gudelunas, et al. presented findings from a laboratory study that demonstrated greater pulse oximetry errors in dark-skinned subjects with low perfusion [21]. While the final version of the Gudelunas, et al. study had not completed peer review at the time of this publication, the preprint paper suggests several key methodology limitations. The study tested legacy (no longer manufactured) devices from Masimo and Nellcor, which could produce errant readings if paired with non-compliant sensors. Also, the methodology did not standardize sensor type or anatomic measurement site, as the Masimo device was tested using two different measurement sites (finger and ear), while the Nellcor device was tested using only the finger. In addition, it is unclear if measurements on different devices were collected simultaneously with two or more sensors on the same subject, which can lead to false readings from crosstalk interference without proper use of optical shielding [26]. Lastly, it is unclear whether the Pi data was properly standardized between the Masimo and Nellcor devices.

We believe the methodology utilized in our paper is scientifically robust; however, there are two notable limitations. First, this study used subjects who self-identified as being racially Black or White, and other ethnic groups (e.g., Asian, Hispanic) were not evaluated. However, study investigators included subjects whose skin pigmentation ranged across the spectrum (Massey Scale values 1 [minimal pigment] through 9 [very dark pigment]). Second, the data were collected from healthy volunteers using a controlled laboratory desaturation protocol; thus, clinical factors that can be observed in critically ill patients, such as tissue edema, anemia and hemoglobinopathies (e.g., sickle cell anemia, thalassemia, etc.) were not represented. However, controlling for these conditions helped minimize confounders that are present in clinical scenarios, allowing for greater focus on the topics of skin tone and Pi. Indeed, abnormal hemoglobin species (e.g., carboxyhemoglobin and methemoglobin) were measured and reported in the earlier paper by Barker and Wilson, and the values were similar (statistically the same) between Black and White groups [20]. Also, one can only ethically conduct desaturation studies using healthy volunteer subjects in a safe setting.

In conclusion, this secondary analysis of data from healthy Black and White volunteers demonstrated that Masimo RD SET® pulse oximeter sensors are accurate in both races during conditions of normal and low peripheral perfusion. Prospective clinical studies are recommended to further elucidate these results.
